# Supplementary material for: Sequential interleukin-17 inhibitors for moderate-to-severe plaque psoriasis who have an IL-17 inhibitors failure in a resource limited country: An economic evaluation
Source: PLoS One. 2024 Aug 9;19(8):e0307050. doi: 10.1371/journal.pone.0307050 (PMC11315331; doi:10.1371/journal.pone.0307050)
Supplement: S1 Table — (PDF) [file pone.0307050.s001.pdf]

S1 Table Cost-utility analysis inputs

Data	Value	Range	Distribution	Reference
**Clinical inputs**				
**Response rate**				
**PASI 75**				
Secukinumab	0.831	0.802 – 0.857	Beta	(1)
Ixekizumab	0.888	0.865 – 0.909	Beta	(1)
Brodalumab	0.887	0.865 – 0.908	Beta	(1)
Guselkumab	0.868	0.838 – 0.894	Beta	(1)
SoC	0.053	0.048 – 0.059	Beta	(1)
**PASI 90**				
Secukinumab	0.614	0.572 – 0.656	Beta	(1)
Ixekizumab	0.708	0.668 – 0.746	Beta	(1)
Brodalumab	0.706	0.668 – 0.746	Beta	(1)
Guselkumab	0.673	0.625 – 0.719	Beta	(1)
SoC	0.011	0.010 – 0.013	Beta	(1)
**PASI 100**				
Secukinumab	0.299	0.263 – 0.339	Beta	(1)
Ixekizumab	0.395	0.352 – 0.440	Beta	(1)
Brodalumab	0.392	0.352 – 0.439	Beta	(1)
Guselkumab	0.357	0.309 – 0.404	Beta	(1)
SoC	0.001	0.001 – 0.001	Beta	(1)
**Dropout rate**				
**Year 1**				
Secukinumab	0.040	0.030 – 0.060	Beta	(2, 3)
Ixekizumab	0.060	0.050 – 0.070	Beta	(4, 5)
Brodalumab	0.020	0.010 – 0.033	Beta	(6)
Guselkumab	0.049	0.031 – 0.068	Beta	(4)
**Year 2 or later**				
Secukinumab	0.050	0.030 – 0.050	Beta	(2, 4)
Ixekizumab	0.031	0.026 – 0.037	Beta	(7)
Brodalumab	0.050	0.025 – 0.100	Beta	(8)
Guselkumab	0.152	0.108 – 0.195	Beta	(9)
**Release rate**				
Secukinumab	0.810	0.780 – 0.840	Beta	(10-12)
Ixekizumab	0.910	0.890 – 0.940	Beta	(7)
Brodalumab	0.940	0.890 – 0.991	Beta	(13)
Guselkumab	0.950	0.625 – 0.759	Beta	(14)
**Adverse event rate**				
**Malignancy**				
Secukinumab	0.009	0.007 – 0.011	Beta	(15)
Ixekizumab	0.005	0.004 – 0.006	Beta	(15)
Brodalumab	0.003	0.002 – 0.004	Beta	(15)
Guselkumab	0.010	0.008 – 0.012	Beta	(15)
**Serious infection**				
Secukinumab	0.016	0.013 – 0.019	Beta	(15)
Ixekizumab	0.032	0.026 – 0.038	Beta	(15)
Brodalumab	0.007	0.006 – 0.008	Beta	(15)
Guselkumab	0.006	0.005 – 0.007	Beta	(15)
**Cirrhosis**				
Methotrexate	0.020	0.008 – 0.031	Beta	Meta-analysis from 8 studies (16-23)
**Utility**				

Data	Value	Range	Distribution	Reference
PASI 75	0.890	0.712 – 1.000	Beta	(24)
PASI 90 – PASI 100	0.906	0.725 – 1.000	Beta	(25)
PASI <75	0.642	0.514 – 0.770	Beta	(8)
Baseline PASI score for Thai patients	15.76	12.41 – 19.11	Beta	(26-28)
Relative risk of death in psoriasis patients	1.520	1.35 – 1.71	Lognormal	(29)
**Resource utilization and cost inputs**				
**Price of biologics and SoC (฿/unit)**				
Secukinumab 150 mg	6,227	N/A	Fixed	DMSIC(30)
Ixekizumab	20,330	N/A	Fixed	DMSIC(30)
Brodalumab	14,980	N/A	Fixed	Company
Guselkumab	61,391	N/A	Fixed	Company
Methotrexate	2.525	N/A	Fixed	DMSIC(30)
Ciclosporin	79.58	N/A	Fixed	DMSIC(30)
**Price of co-treatments (฿/unit)**				
LCD 450 g		N/A	Fixed	DMSIC(30)
Tar shampoo 200 ml		N/A	Fixed	DMSIC(30)
Calcipotriol 30 g		N/A	Fixed	DMSIC(30)
Betamethasone cream 500 g		N/A	Fixed	DMSIC(30)
% Use of methotrexate	0.854	0.852 – 0.856	Beta	(31)
**% Use of Co-treatments**				
LCD	0.805	0.780 – 0.829	Beta	(31)
Tar shampoo	0.805	0.780 – 0.829	Beta	(31)
Calcipotriol	0.179	0.155 – 0.202	Beta	(31)
Betamethasone cream	0.960	0.948 – 0.972	Beta	(31)
**Screening cost (one-time cost; ฿/unit)**				
IGRA	2,122	1,698 – 2,546	Gamma	(32)
Chest X-ray	255	204 – 306	Gamma	Standard cost list(33)
AST	75	60 – 89	Gamma	Standard cost list(33)
ALT	75	60 – 89	Gamma	Standard cost list(33)
CBC	135	108 – 162	Gamma	Standard cost list(33)
HBsAg	195	156 – 243	Gamma	Standard cost list(33)
Anti-HBs	270	216 – 324	Gamma	Standard cost list(33)
Anti-HBc	345	276 – 414	Gamma	Standard cost list(33)
**Monitoring cost (annual cost, ฿/unit)**				
QuantiFERON	2,122	1,698 – 2,546	Gamma	(32)
CBC	269	215 – 323	Gamma	Standard cost list(33)
AST	149	119 – 179	Gamma	Standard cost list(33)
ALT	149	119 – 179	Gamma	Standard cost list(33)
BUN	149	119 – 179	Gamma	Standard cost list(33)
Serum creatinine	149	119 – 179	Gamma	Standard cost list(33)
Lipid profile	1,202	961 – 1,442	Gamma	Standard cost list(33)
Uric acid	360	288 – 433	Gamma	Standard cost list(33)
Electrolyte	481	384 – 577	Gamma	Standard cost list(33)
Magnesium	418	335 – 502	Gamma	Standard cost list(33)
**Adverse event cost (annual cost, ฿/year)**				
Malignancy	32,431	25,644 – 38,917	Gamma	(34)

Data	Value	Range	Distribution	Reference
Serious infection	92,845	74,276 – 111,414	Gamma	(34)
Cirrhosis	90,702	72,561 – 108,842	Gamma	(35)
Transportation cost (฿/visit)	159	133 – 184	Gamma	Standard cost list(33)
Additional food cost (฿/visit)	58	47 – 70	Gamma	Standard cost list(33)
Number of visits per year	3.84	3.07 – 4.61	Beta	(36)

Abbreviation: AST; aspartate aminotransferase, ALT; alanine transaminase, Anti-HBs; hepatitis B surface antibody, Anti-HBc; hepatitis B core antibody, BUN; blood urea nitrogen, CBC; complete blood count, DMSIC; Drug and Medical Supply Information Center, Ministry of Public Health**,** HBsAg; hepatis B surface antigen**,** LCD; liquor carbonis detergents

References

1. Armstrong AW, Puig L, Joshi A, Skup M, Williams D, Li J, et al. Comparison of Biologics and Oral Treatments for Plaque Psoriasis: A Meta-analysis. JAMA Dermatol. 2020;156(3):258-69.
2. Bissonnette R, Luger T, Thaci D, Toth D, Messina I, You R, et al. Secukinumab sustains good efficacy and favourable safety in moderate-to-severe psoriasis after up to 3 years of treatment: results from a double-blind extension study. Br J Dermatol. 2017;177(4):1033-42.
3. Langley RG, Elewski BE, Lebwohl M, Reich K, Griffiths CE, Papp K, et al. Secukinumab in plaque psoriasis--results of two phase 3 trials. N Engl J Med. 2014;371(4):326-38.
4. Blauvelt A, Papp K, Gottlieb A, Jarell A, Reich K, Maari C, et al. A head-to-head comparison of ixekizumab vs. guselkumab in patients with moderate-to-severe plaque psoriasis: 12-week efficacy, safety and speed of response from a randomized, double-blinded trial. Br J Dermatol. 2020;182(6):1348-58.
5. Griffiths CE, Reich K, Lebwohl M, van de Kerkhof P, Paul C, Menter A, et al. Comparison of ixekizumab with etanercept or placebo in moderate-to-severe psoriasis (UNCOVER-2 and UNCOVER-3): results from two phase 3 randomised trials. Lancet. 2015;386(9993):541-51.
6. Papp KA, Reich K, Paul C, Blauvelt A, Baran W, Bolduc C, et al. A prospective phase III, randomized, double-blind, placebo-controlled study of brodalumab in patients with moderate-to-severe plaque psoriasis. Br J Dermatol. 2016;175(2):273-86.
7. Gordon KB, Blauvelt A, Papp KA, Langley RG, Luger T, Ohtsuki M, et al. Phase 3 Trials of Ixekizumab in Moderate-to-Severe Plaque Psoriasis. N Engl J Med. 2016;375(4):345-56.
8. Hendrix N, Ollendorf DA, Chapman RH, Loos A, Liu S, Kumar V, et al. Cost-Effectiveness of Targeted Pharmacotherapy for Moderate to Severe Plaque Psoriasis. J Manag Care Spec Pharm. 2018;24(12):1210-7.
9. Lytvyn Y, Zaaroura H, Mufti A, AlAbdulrazzaq S, Yeung J. Drug survival of guselkumab in patients with plaque psoriasis: A 2 year retrospective, multicenter study. JAAD Int. 2021;4:49-51.
10. Blauvelt A, Reich K, Warren RB, Szepietowski JC, Sigurgeirsson B, Tyring SK, et al. Secukinumab re-initiation achieves regain of high response levels in patients who interrupt treatment for moderate to severe plaque psoriasis. Br J Dermatol. 2017;177(3):879-81.
11. Mrowietz U, Leonardi CL, Girolomoni G, Toth D, Morita A, Balki SA, et al. Secukinumab retreatment-as-needed versus fixed-interval maintenance regimen for moderate to severe plaque psoriasis: A randomized, double-blind, noninferiority trial (SCULPTURE). J Am Acad Dermatol. 2015;73(1):27-36 e1.
12. Lebwohl M, Iversen L, Eidsmo L, Messina I, You R, Milutinovic M. Long-term psoriasis control following secukinumab discontinuation indicates disease modification of moderate to severe psoriasis. J Clin Aesthet Dermatol. 2017;10(5):S28 - S9.
13. Papp K, Menter A, Leonardi C, Soung J, Weiss S, Pillai R, et al. Long-term efficacy and safety of brodalumab in psoriasis through 120 weeks and after withdrawal and retreatment: subgroup analysis of a randomized phase III trial (AMAGINE-1). Br J Dermatol. 2020;183(6):1037-48.

14. Gordon KB, Armstrong AW, Foley P, Song M, Shen YK, Li S, et al. Guselkumab Efficacy after Withdrawal Is Associated with Suppression of Serum IL-23-Regulated IL-17 and IL-22 in Psoriasis: VOYAGE 2 Study. J Invest Dermatol. 2019;139(12):2437-46 e1.
15. Loft ND, Vaengebjerg S, Halling AS, Skov L, Egeberg A. Adverse events with IL-17 and IL-23 inhibitors for psoriasis and psoriatic arthritis: a systematic review and meta-analysis of phase III studies. J Eur Acad Dermatol Venereol. 2020;34(6):1151-60.
16. Berends MA, Snoek J, de Jong EM, van de Kerkhof PC, van Oijen MG, van Krieken JH, et al. Liver injury in long-term methotrexate treatment in psoriasis is relatively infrequent. Aliment Pharmacol Ther. 2006;24(5):805-11.
17. Malatjalian DA, Ross JB, Williams CN, Colwell SJ, Eastwood BJ. Methotrexate hepatotoxicity in psoriatics: report of 104 patients from Nova Scotia, with analysis of risks from obesity, diabetes and alcohol consumption during long term follow-up. Can J Gastroenterol. 1996;10(6):369-75.
18. Mitchell D, Smith A, Rowan B, Warnes TW, Haboubi NY, Lucas SB, et al. Serum type III procollagen peptide, dynamic liver function tests and hepatic fibrosis in psoriatic patients receiving methotrexate. Br J Dermatol. 1990;122(1):1-7.
19. Reese LT, Grisham JW, Aach RD, Eisen AZ. Effects of methotrexate on the liver in psoriasis. J Invest Dermatol. 1974;62(6):597-602.
20. Rosenberg P, Urwitz H, Johannesson A, Ros AM, Lindholm J, Kinnman N, et al. Psoriasis patients with diabetes type 2 are at high risk of developing liver fibrosis during methotrexate treatment. J Hepatol. 2007;46(6):1111-8.
21. Themido R, Loureiro M, Pecegueiro M, Brandao M, Campos MC. Methotrexate hepatotoxicity in psoriatic patients submitted to long-term therapy. Acta Derm Venereol. 1992;72(5):361-4.
22. Van Dooren-Greebe RJ, Kuijpers AL, Mulder J, De Boo T, Van de Kerkhof PC. Methotrexate revisited: effects of long-term treatment in psoriasis. Br J Dermatol. 1994;130(2):204-10.
23. Zachariae H, Kragballe K, Sogaard H. Methotrexate induced liver cirrhosis. Studies including serial liver biopsies during continued treatment. Br J Dermatol. 1980;102(4):407-12.
24. Sun HY, Keller E, Suresh H, Sebaratnam DF. Biologics for severe, chronic plaque psoriasis: An Australian cost-utility analysis. JAAD Int. 2021;5:1-8.
25. Igarashi A, Igarashi A, Graham CN, Gilloteau I, Tani Y. Evaluating the cost-effectiveness of secukinumab in moderate-to-severe psoriasis: a Japanese perspective. J Med Econ. 2018:1-9.
26. Kongthong S, Phumyen A, Meephansan J. Effect of narrowband ultraviolet B therapy on serum levels of CD26/dipeptidyl-peptidase IV and truncated forms of substance P in psoriasis patients with pruritus. Clin Cosmet Investig Dermatol. 2019;12:597-604.
27. Leenutaphong V, Nimkulrat P, Sudtim S. Comparison of phototherapy two times and four times a week with low doses of narrow-band ultraviolet B in Asian patients with psoriasis. Photodermatol Photoimmunol Photomed. 2000;16(5):202-6.
28. Silpa-Archa N, Pattanaprichakul P, Charoenpipatsin N, Jansuwan N, Udompunthurak S, Chularojanamontri L, et al. The efficacy of UVA1 phototherapy in psoriasis: Clinical and histological aspects. Photodermatol Photoimmunol Photomed. 2020;36(1):21-8.
29. Dhana A, Yen H, Yen H, Cho E. All-cause and cause-specific mortality in psoriasis: A systematic review and meta-analysis. J Am Acad Dermatol. 2019;80(5):1332-43.
30. Drug and Medical Supply Information Center: Minsitry of Public Health. Reference price Nonthaburi, Thailand: Ministry of Public Health; 2021 [Available from: http://dmsic.moph.go.th/index/drugsearch/3.
31. Jiamton S, Suthipinittharm P, Kulthanan K, Chularojanamontri L, Wongpraparut C, Silpa-archa N, et al. Clinical characteristics of Thai patients with psoriasis. J Med Assoc Thai. 2012;95(6):795-801.
32. Srriwijitalai W, Wiwanitkit V. Cost-effectiveness analysis of common tuberculosis screening laboratory tests for hemodialysis patients: An analysis from tropical endemic country, Thailand. Saudi J Kidney Dis Transpl. 2018;29(2):476-7.
33. Health Intervention and Technology Assessment Program; Ministry of Public Health. Standard cost list for health technology assessment Nonthaburi, Thailand: Ministry of Public Health 2010 [Available from: https://costingmenu.hitap.net/.
34. Osiri M, Dilokthornsakul P, Chokboonpium S, Suthipinijtham P, Koolvisoot A. Budget Impact of Sequential Treatment with Biologics, Biosimilars, and Targeted Synthetic Disease-Modifying Antirheumatic Drugs in Thai Patients with Rheumatoid Arthritis. Adv Ther. 2021;38(9):4885-99.

35. Dilokthornsakul P, Sawangjit R, Tangkijvanich P, Chayanupatkul M, Tanwandee T, Sukeepaisarnjaroen W, et al. Economic Evaluation of Oral Nucleos(t)ide Analogues for Patients with Chronic Hepatitis B in Thailand. Appl Health Econ Health Policy. 2022.
36. Chaiyamahapruk S, Warnnissorn P. Prevalence and characteristics of psoriasis patients in a primary care area in Thailand. J Med Assoc Thai. 2021;104(4):610 - 4.
