# Supplementary material for: Sequential interleukin-17 inhibitors for moderate-to-severe plaque psoriasis who have an IL-17 inhibitors failure in a resource limited country: An economic evaluation
Source: PLoS One. 2024 Aug 9;19(8):e0307050. doi: 10.1371/journal.pone.0307050 (PMC11315331; doi:10.1371/journal.pone.0307050)
Supplement: S2 Table — (PDF) [file pone.0307050.s002.pdf]

S2 Table Budget impact analysis inputs

Data	Value	Reference
Thai population aged ≥ 40	31,629,232	National Statistics Office (1)
Prevalence of psoriasis	0.133%	(2)
Incidence of psoriasis	0.030%	(3)
%Death	0.531%	Calculated from CEA model
%Systemic treatment	33.50%	(4)
%Uptake per year	5.00%	Assumption
Average relapse rate	91.00%	Calculated from CEA model

References

1. National Statistical Office Thailand. Number of Population from Registration by Age, Sex, Region and Province: 2021 Bangkok: National Statistical Office Thailand; 2021 [
2. Chaiyamahapruk S, Warnnissorn P. Prevalence and characteristics of psoriasis patients in a primary care area in Thailand. J Med Assoc Thai. 2021;104(4):610 - 4.
3. Parisi R, Iskandar IYK, Kontopantelis E, Augustin M, Griffiths CEM, Ashcroft DM, et al. National, regional, and worldwide epidemiology of psoriasis: systematic analysis and modelling study. BMJ. 2020;369:m1590.
4. Jiamton S, Suthipinittharm P, Kulthanan K, Chularojanamontri L, Wongpraparut C, Silpa-archa N, et al. Clinical characteristics of Thai patients with psoriasis. J Med Assoc Thai. 2012;95(6):795-801.
