# Supplementary material for: Sequential interleukin-17 inhibitors for moderate-to-severe plaque psoriasis who have an IL-17 inhibitors failure in a resource limited country: An economic evaluation
Source: PLoS One. 2024 Aug 9;19(8):e0307050. doi: 10.1371/journal.pone.0307050 (PMC11315331; doi:10.1371/journal.pone.0307050)
Supplement: S3 Table — (PDF) [file pone.0307050.s003.pdf]

S3 Table Budget impact analysis findings

Analysis	Year 1	Year 2	Year 3	Year 4	Year 5	Average (THB/year)
**Cost estimation (THB)**						
Standard of Care	239,247,989	283,281,361	327,314,733	371,348,105	415,381,477	327,314,733
Sequence 1	392,298,757	462,565,812	508,027,729	431,558,516	589,811,238	476,852,410
Sequence 2	504,619,358	549,161,781	614,632,258	477,946,787	700,496,217	569,371,280
Sequence 3	535,214,972	644,833,625	734,261,851	518,081,071	825,512,673	651,580,839
**Budget impact analysis (THB)**						
Sequence 1	153,050,768	179,284,451	180,712,996	60,210,411	174,429,761	149,537,677
Sequence 2	265,371,370	265,880,420	287,317,525	106,598,682	285,114,740	242,056,547
Sequence 3	295,966,984	361,552,264	406,947,118	146,732,966	410,131,196	324,266,105

Abbreviation: THB; Thai baht

Note: Sequence 1: Secukinumab followed by Ixekizumab/Brodalumab then Guselkumab, Sequence 2: Ixekizumab followed by Secukinumab /Brodalumab then Guselkumab, Sequence 3: Brodalumab followed by Ixekizumab/Secukinumab then Guselkumab
