# Supplementary material for: Sequential interleukin-17 inhibitors for moderate-to-severe plaque psoriasis who have an IL-17 inhibitors failure in a resource limited country: An economic evaluation
Source: PLoS One. 2024 Aug 9;19(8):e0307050. doi: 10.1371/journal.pone.0307050 (PMC11315331; doi:10.1371/journal.pone.0307050)
Supplement: S1 Fig — (PDF) [file pone.0307050.s004.pdf]

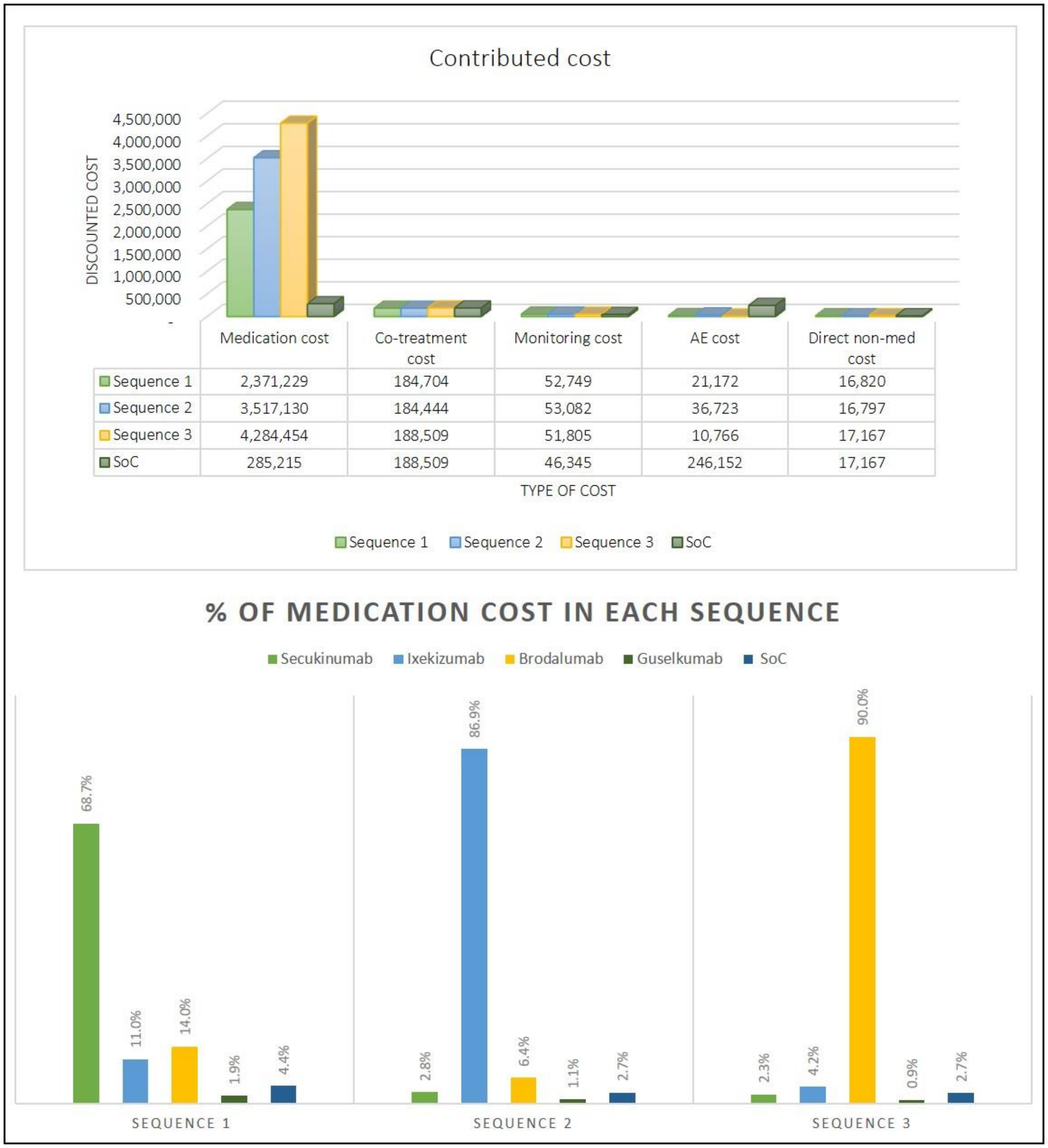

S1 Fig. Cost contribution (A) % of cost contribution for each sequence, (B) % of cost of each treatment within each seqeunce
