# Supplementary figures and images for: Sequential interleukin-17 inhibitors for moderate-to-severe plaque psoriasis who have an IL-17 inhibitors failure in a resource limited country: An economic evaluation
Source: PLoS One. 2024 Aug 9;19(8):e0307050. doi: 10.1371/journal.pone.0307050 (PMC11315331; doi:10.1371/journal.pone.0307050)

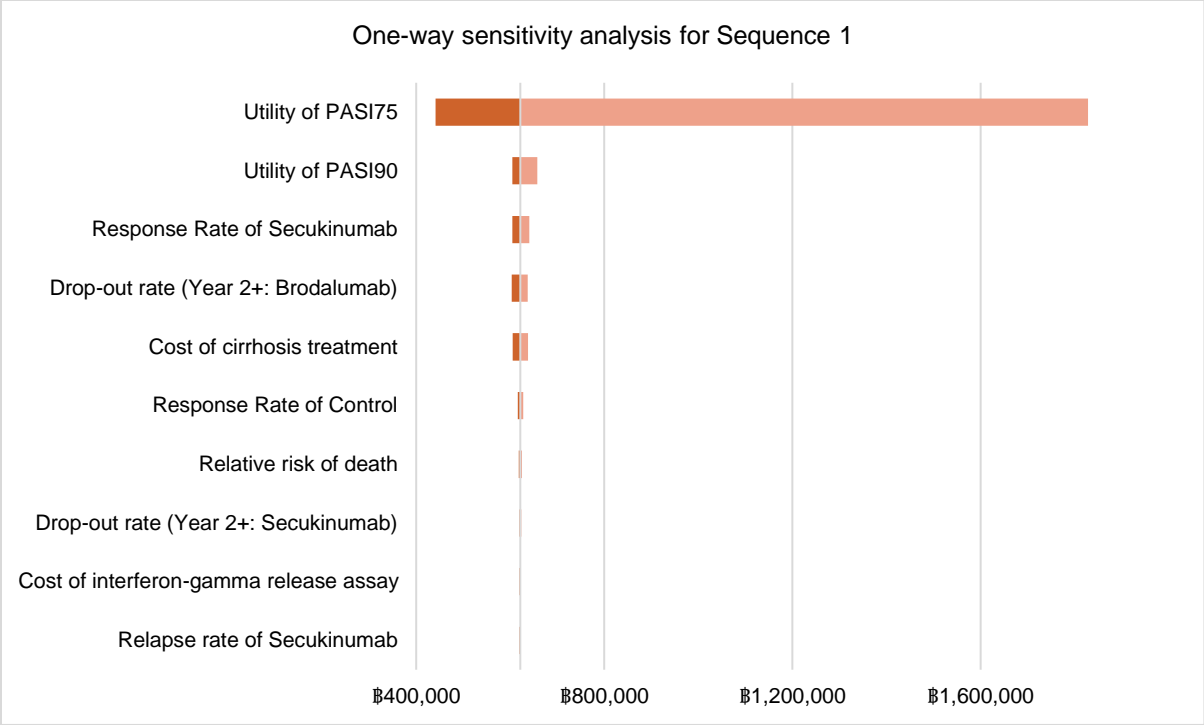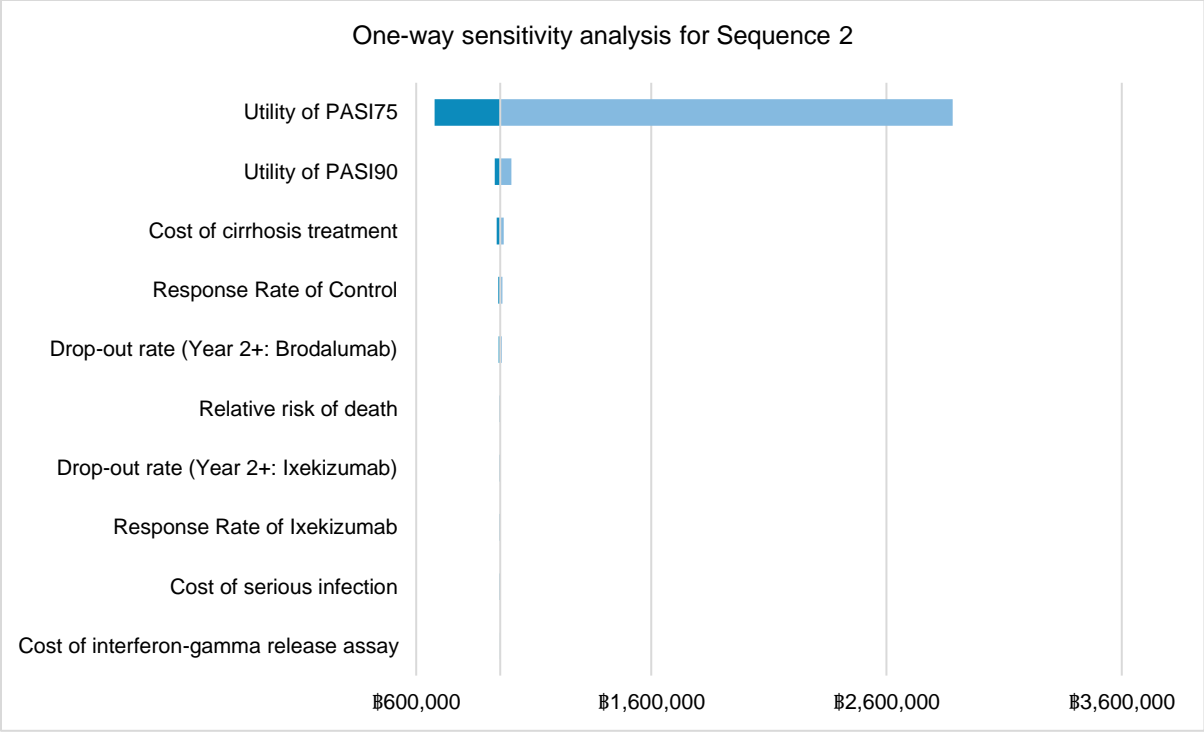

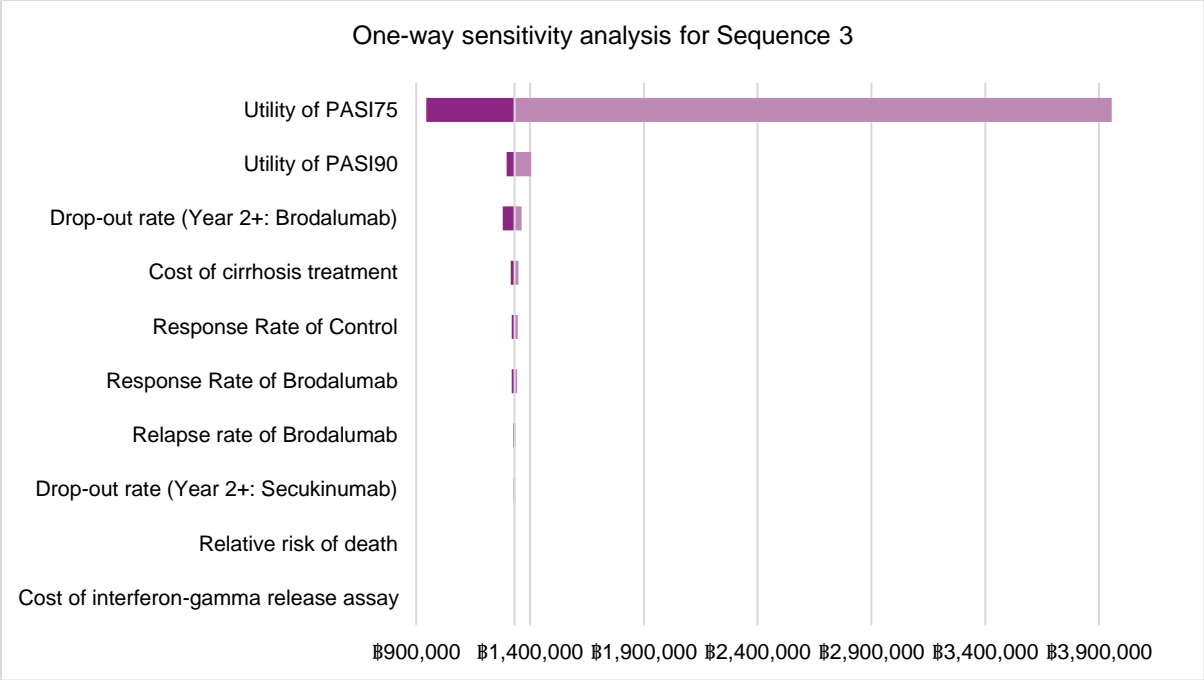

S2 Fig. Tornado plots

Supplement: S2 Fig — (PDF) [file pone.0307050.s005.pdf]

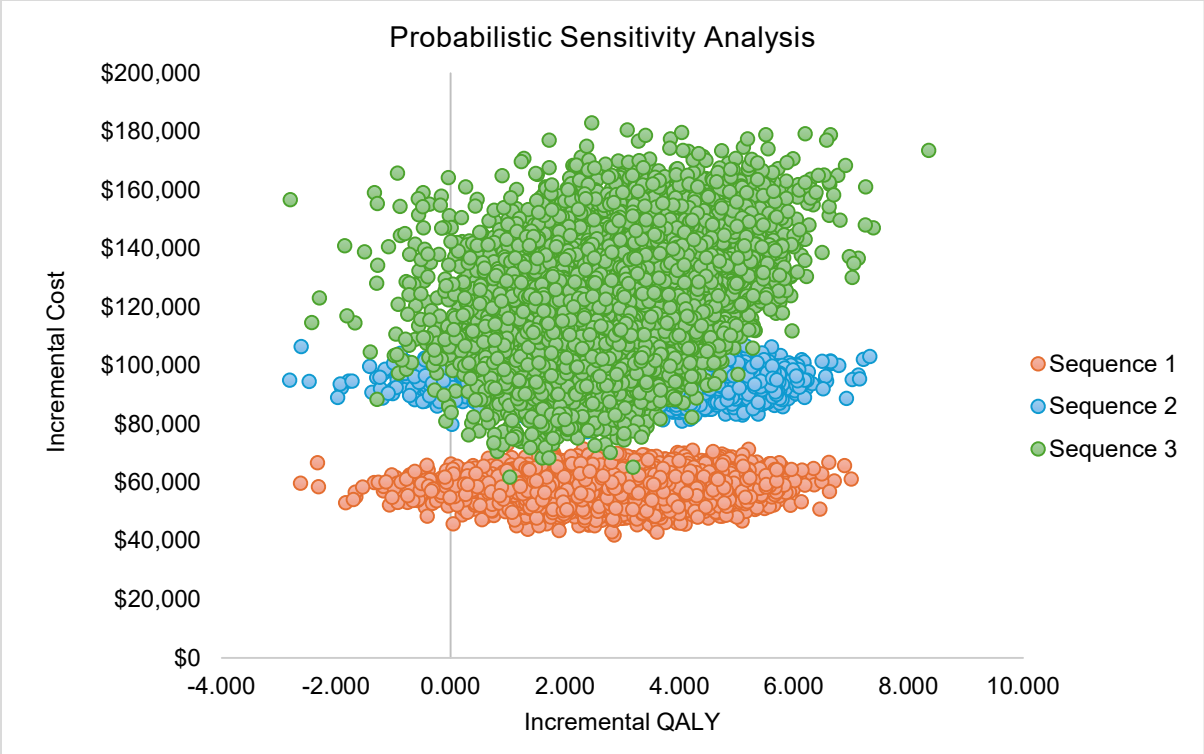

S3 Fig. Cost-effectiveness analysis plane

Supplement: S3 Fig — (PDF) [file pone.0307050.s006.pdf]
