# Supplementary material for: Sequential interleukin-17 inhibitors for moderate-to-severe plaque psoriasis who have an IL-17 inhibitors failure in a resource limited country: An economic evaluation
Source: PLoS One. 2024 Aug 9;19(8):e0307050. doi: 10.1371/journal.pone.0307050 (PMC11315331; doi:10.1371/journal.pone.0307050)
Supplement: Attachment — Submitted filename: A rebuttal letter R1 v2.docx [file pone.0307050.s007.docx]

**A rebuttal letter responding to reviewers’ comments point-by-point for a manuscript entitled** **“Sequential interleukin-17 inhibitors for moderate-to-severe plaque psoriasis who have an inadequate response to systemic conventional treatments in a resource limited country: an economic evaluation”**

When submitting your revision, we need you to address these additional requirements.

**Comment 1**

1. Please ensure that your manuscript meets PLOS ONE's style requirements, including those for file naming. The PLOS ONE style templates can be found at 
<https://journals.plos.org/plosone/s/file?id=wjVg/PLOSOne_formatting_sample_main_body.pdf> and 
<https://journals.plos.org/plosone/s/file?id=ba62/PLOSOne_formatting_sample_title_authors_affiliations.pdf>.

**Response:**

We revised the manuscript format according to the guidelines throughout the manuscript.

**Comment 2**

2. Please note that PLOS ONE has specific guidelines on code sharing for submissions in which author-generated code underpins the findings in the manuscript. In these cases, all author-generated code must be made available without restrictions upon publication of the work. Please review our guidelines at <https://journals.plos.org/plosone/s/materials-and-software-sharing#loc-sharing-code> and ensure that your code is shared in a way that follows best practice and facilitates reproducibility and reuse.

**Response:**

We have made our model available in the Harvard Dataverse network at this link. <https://doi.org/10.7910/DVN/HN2K7P>

**Comment 3**

3. Thank you for stating the following in the Competing Interests section: 
[Piyameth Dilokthornsakul receives research grants from Novartis (Thailand) Limited, Pfizer (Thailand) Limited. He also receives honorariums from GSK (Thailand) Limited, and Boehringer Ingelheim (Thailand) Limited. Other authors declare no conflict of interest.].

Please confirm that this does not alter your adherence to all PLOS ONE policies on sharing data and materials, by including the following statement: ""This does not alter our adherence to  PLOS ONE policies on sharing data and materials.” (as detailed online in our guide for authors <http://journals.plos.org/plosone/s/competing-interests>).  If there are restrictions on sharing of data and/or materials, please state these. Please note that we cannot proceed with consideration of your article until this information has been declared.

Please include your updated Competing Interests statement in your cover letter; we will change the online submission form on your behalf.

**Response 3**

We confirmed that our conflict of interest does not later our adherence to PLOS ONE policies on sharing data and materials.

We added it in the Competing interest statement section as shown below.

This does not alter our adherence to PLOS ONE policies on sharing data and materials. Other authors declare no conflict of interest.

**Comment 4**

4. We note that you have indicated that there are restrictions to data sharing for this study. PLOS only allows data to be available upon request if there are legal or ethical restrictions on sharing data publicly. For more information on unacceptable data access restrictions, please see <http://journals.plos.org/plosone/s/data-availability#loc-unacceptable-data-access-restrictions>.

Before we proceed with your manuscript, please address the following prompts:

a) If there are ethical or legal restrictions on sharing a de-identified data set, please explain them in detail (e.g., data contain potentially identifying or sensitive patient information, data are owned by a third-party organization, etc.) and who has imposed them (e.g., a Research Ethics Committee or Institutional Review Board, etc.). Please also provide contact information for a data access committee, ethics committee, or other institutional body to which data requests may be sent.

b) If there are no restrictions, please upload the minimal anonymized data set necessary to replicate your study findings to a stable, public repository and provide us with the relevant URLs, DOIs, or accession numbers. For a list of recommended repositories, please see
<https://journals.plos.org/plosone/s/recommended-repositories>. You also have the option of uploading the data as Supporting Information files, but we would recommend depositing data directly to a data repository if possible.

We will update your Data Availability statement on your behalf to reflect the information you provide.

**Response 4**

We have made our model available in the Harvard Dataverse network at this link. <https://doi.org/10.7910/DVN/HN2K7P>

We revised the Data sharing statement as shown below.

Data used in this study is available at <https://doi.org/10.7910/DVN/HN2K7P>

**Comment 5**

5. In the online submission form, you indicated that [Data used in this study will be available by appropriate request to the corresponding author. Only data could be provided for non-commercial purposes.]. 
All PLOS journals now require all data underlying the findings described in their manuscript to be freely available to other researchers, either 1. In a public repository, 2. Within the manuscript itself, or 3. Uploaded as supplementary information.
This policy applies to all data except where public deposition would breach compliance with the protocol approved by your research ethics board. If your data cannot be made publicly available for ethical or legal reasons (e.g., public availability would compromise patient privacy), please explain your reasons on resubmission and your exemption request will be escalated for approval.

**Response 5**

We have made our model available in the Harvard Dataverse network at this link. <https://doi.org/10.7910/DVN/HN2K7P>

We revised the Data sharing statement as shown below.

Data used in this study is available at <https://doi.org/10.7910/DVN/HN2K7P>

**Comment 6**

6. Please include captions for your Supporting Information files at the end of your manuscript, and update any in-text citations to match accordingly. Please see our Supporting Information guidelines for more information: <http://journals.plos.org/plosone/s/supporting-information>.

[Note: HTML markup is below. Please do not edit.]

**Response 6**

We revised our manuscript and supplement files according to the guideline.

**Comments to the Author**
**Reviewer #1:**

**Comment 7**

The title of the article need to be improved.
**Response 7**

We revised our title to make in more concise as shown below.

**Original version**

Sequential interleukin-17 inhibitors for moderate-to-severe plaque psoriasis who have an inadequate response to systemic conventional treatments in a resource limited country: an economic evaluation

**Revised version**

Sequential interleukin-17 inhibitors for moderate-to-severe plaque psoriasis who previously have an IL-17 inhibitors failure in a resource limited country: an economic evaluation

**Comment 8**
-A main concern is that the manuscript was funded Funding statement:
This work was supported by Novartis (Thailand) Limited. it maybe possible that the Principal Investigator who has the conflicts of interest could be indirectly helping Novartis medication Secukinumab to be approved in Thailand.
**Response 8**

We conducted this study based on the scientific merit not commercial interest. Even though the study was funded by Novartis (Thailand), the funder did not alter our study design, data collection, data analysis, or manuscript writing. The funder only reviewed and approved the submission without any major correction. We originally declared them in the Role of the Funder section. Thus, we don’t think we were attempting to helping secukinumab to be approved in Thailand but providing scientific information on the cost-effectiveness of sequential treatments of anti-IL17 for psoriasis in Thailand for both clinical and policy decisions.

**Comment 9**
The reviewer RESPECTFULLY RECOMMEND TO REQUEST AN OPINION WITH AN STATISTICIAN TO REVIEW THE METHODOLOGY THAT IS SOMEHOW CONFUSING.

**Response 9**

To better explain our statistical analysis, we revised our data analysis section to better explanation as shown below.

**Original version**

**2.6.1 Cost-effective analysis**

Base-case analysis was performed, and the incremental cost-effectiveness ratio (ICER) was calculated. All sequential IL-17 inhibitors were compared to SoC. The fully incremental analysis was also performed to compare quality-adjusted life-years (QALYs) and costs among sequential IL-17 inhibitor treatments. An annual discount rate of 3% was applied.[12, 13]

A series of one-way sensitivity analysis was conducted to explore uncertainties among inputs. The 95% credible interval of each ICER and cost-effectiveness acceptability curve were estimated based on the 10,000 iterations of Monte Carlo simulation. The willingness-to-pay (WTP) threshold of 160,000 THB/QALY (5,008 $/QALY) was used.[12, 13]

Scenario analyses were also performed including: (1) the changes of information sources of health utility value from previous cost-effectiveness studies to the calculation from pooled baseline PASI in Thai patients, (2 - 3) changes of the definition of treatment response from PASI 75 to PASI 90 and PASI 100, (4-7) changes in a ratio between MTX and ciclosporin from 85:15 to 80:20, 70:30, 0:100, and 100:0 for SoC, (8) changes in MTX from tablet form to injection. All scenario analyses were recommended by experts.

**2.6.2 Budget impact analysis**

A budget impact model was developed to estimate the financial consequences of the adoption of sequential IL-17 inhibitors in lieu of SoC over 5-year time horizon under a payer’s perspective. The dynamic cohort model was used to estimate the number of eligible patients for sequential IL-17 inhibitors. The estimated number of populations aged 40 years or older was approximately 31.6 million.[37] Prevalence,[36] incidence,[38] proportion of patients receiving systemic treatments,[14] and average annual death rate were applied. A 5% uptake rate was assumed. The 91% relapse rate was used for year 4-5 because all patients were assumed to stop their biologic treatments at the end of year 3. All inputs used in budget impact analysis are presented in supplement at **S2 Table**.

**Revised version**

**2.6.1 Cost-effectiveness analysis**

The incremental cost-effectiveness ratio (ICER) using mean or point estimates of each input was calculated as base-case analysis to compare all sequential IL-17 inhibitors with SoC using the formula.

ICER = Total discounted cost of sequential IL-17 inhibitors - Total discounted cost of SoC

Total discounted QALY of sequential IL-17 inhibitors - Total discounted QALY of SoC

The fully incremental analysis was also performed to calculate the ICER of each comparison between sequential IL-17 inhibitors. All analyses applied an annual discount rate of 3% for both cost and QALY.[12, 13] The willingness-to-pay (WTP) of 160,000 THB/QALY (5,008 $/QALY) was used as the cost-effectiveness threshold.[12, 13]

A series of one-way sensitivity analysis was conducted to explore the effect of uncertainties around inputs on ICER. The upper and lower bound of 95% confidence intervals or ± 20% from mean or point estimates of each input were utilized in the one-way sensitivity analyses and presented the findings as tornado plots.

Probabilistic sensitivity analysis was also conducted to explore the effect of uncertainties around inputs on ICER using Monte Carlo simulation. The Probabilistic sensitivity analysis randomly selected the value of each input simultaneously and calculated ICER of each iteration for 10,000 iterations and showed the findings as cost-effectiveness analysis plane. The 95% credible interval of ICER from the simulation was also calculated. In addition, the cost-effectiveness acceptability curve by varying WTP was created.

Additionally, scenario analyses were also performed including: (1) the changes of information sources of health utility value from previous cost-effectiveness studies to the calculation from pooled baseline PASI in Thai patients, (2 - 3) changes of the definition of treatment response from PASI 75 to PASI 90 and PASI 100, (4-7) changes in a ratio between MTX and ciclosporin from 85:15 to 80:20, 70:30, 0:100, and 100:0 for SoC, (8) changes in MTX from tablet form to injection. All scenario analyses were recommended by experts.

**2.6.2 Budget impact analysis**

A budget impact model was developed to estimate the financial consequences of the adoption of sequential IL-17 inhibitors in lieu of SoC over 5-year time horizon under a payer perspective. The dynamic cohort model was employed to estimate the number of eligible patients for sequential IL-17 inhibitors. The estimated number of populations aged 40 years or older was approximately 31.6 million.[37] Prevalence,[36] incidence,[38] proportion of patients receiving systemic treatments,[14] and average annual death rate were applied. A 5% uptake rate was assumed. The 91% relapse rate was used for year 4-5 because all patients were assumed to stop their biologic treatments at the end of year 3. All inputs used in budget impact analysis are presented in supplement at **S2 Table**.

**Reviewer #2: Title**

**Comment 10**
Correction in title: plaque and not plague
**Response 10**

We edited it as suggested.

**Comment 11**
Methods
Explain the ethical issues of the study in the methods, including approval by a research ethics committee
**Response 11**

Because this study was model-based economic evaluation. All inputs were from existing literature. No human subjects are involved. Thus, EC approval is not required. We add it in our method as shown below.

**Original version**

(none)

**Revised version**

This study was model-based economic evaluation using all data from existing literature without any additional primary data collection or human subject involvement. Thus, ethical committee approval is not required.

**Comment 11**
Conclusion
I think the conclusions are poor. The results show other things which could be added as conclusions.

**Response 11**

We revised our conclusion to better conclude our findings as shown below.

**Original version**

At the current willingness-to-pay, no sequential IL17 inhibitor was cost-effective compared to SoC in Thailand.

**Revised version**

At the current willingness-to-pay, no sequential IL17 inhibitor was cost-effective compared to SoC in Thailand. The possibility of being cost-effective for all sequential treatments was very low compared to SoC. Secukinumab followed by ixekizumab or brodalumab then guselkumab (Sequence 1) seems to be the better option compared with other sequential treatments. Policy makers might need to create strategies to control IL-17 inhibitors price and improve the accessibility to all IL-17 inhibitors as a sequential treatment for patients with moderate-to-severe plaque psoriasis who previously failed one of IL-17 inhibitors in a real-world setting.
